# Differential Expression of Melanopsin Isoforms Opn4L and Opn4S during Postnatal Development of the Mouse Retina

**DOI:** 10.1371/journal.pone.0034531

**Published:** 2012-04-05

**Authors:** Steven Hughes, Laura Welsh, Christiana Katti, Irene González-Menéndez, Michael Turton, Stephanie Halford, Sumathi Sekaran, Stuart N. Peirson, Mark W. Hankins, Russell G. Foster

**Affiliations:** 1 Nuffield Laboratory of Ophthalmology, University of Oxford, Oxford, United Kingdom; 2 Departamento de Morfología y Biología Celular, Universidad de Oviedo, Oviedo, Spain; Vanderbilt University, United States of America

## Abstract

Photosensitive retinal ganglion cells (pRGCs) respond to light from birth and represent the earliest known light detection system to develop in the mouse retina. A number of morphologically and functionally distinct subtypes of pRGCs have been described in the adult retina, and have been linked to different physiological roles. We have previously identified two distinct isoforms of mouse melanopsin, Opn4L and Opn4S, which are generated by alternate splicing of the *Opn4* locus. These isoforms are differentially expressed in pRGC subtypes of the adult mouse retina, with both Opn4L and Opn4S detected in M1 type pRGCs, and only Opn4L detected in M2 type pRGCs. Here we investigate the developmental expression of Opn4L and Opn4S and show a differential profile of expression during postnatal development. *Opn4S* mRNA is detected at relatively constant levels throughout postnatal development, with levels of Opn4S protein showing a marked increase between P0 and P3, and then increasing progressively over time until adult levels are reached by P10. By contrast, levels of *Opn4L* mRNA and protein are low at birth and show a marked increase at P14 and P30 compared to earlier time points. We suggest that these differing profiles of expression are associated with the functional maturation of M1 and M2 subtypes of pRGCs. Based upon our data, Opn4S expressing M1 type pRGCs mature first and are the dominant pRGC subtype in the neonate retina, whereas increased expression of Opn4L and the maturation of M2 type pRGCs occurs later, between P10 and P14, at a similar time to the maturation of rod and cone photoreceptors. We suggest that the distinct functions associated with these cell types will develop at different times during postnatal development.

## Introduction

Melanopsin expressing retinal ganglion cells are photosensitive (pRGCs) and represent a third class of ocular photoreceptor involved in the regulation of irradiance detection and non-image forming responses to light, including pupil constriction, circadian entrainment and the regulation of sleep [1], [2]. In mice, pRGCs are photosensitive from birth and are the earliest light detection system to develop in the mammalian retina [3], [4], [5]. However, it is now clear that multiple subtypes of pRGCs exist in the adult mammalian retina [6]. These pRGC subtypes are characterised based primarily on levels of melanopsin expression and the stratification of their dendrites within specific sub laminae of the inner plexiform layer (IPL). M1 type pRGCs express higher levels of melanopsin and have dendrites located in the OFF layer of the IPL, whereas M2 type pRGCs have lower levels of melanopsin expression and dendrites that stratify in the ON sub lamina of the IPL [7], [8], [9], [10], [11], [12]. A third type of pRGC, termed M3 type pRGCs, has also been described with dendrites in both the OFF and ON layers of the IPL [7], [10], [12], [13], but these cells are rare and may represent an anomalous class of pRGC [10], [13]. Most recently two further pRGC subtypes have been identified; M4 and M5 type pRGCs, that are broadly similar in morphology to M2 type pRGCs with dendrites stratifying in the ON layer of the IPL. However, levels of melanopsin expression are low in these cells and they are not easily identified using a highly sensitive melanopsin antibody [8], [10].

In addition to their distinctive morphology and retinal connections there is growing evidence that functional differences exist between the pRGC subtypes, including membrane properties and resting membrane potentials, as well as levels of photosensitivity and the kinetics of photoresponses [8], [14], [15]. Most notable is the observation that the pRGC subtypes innervate specific retino-recipient brain areas [11], [16], [17] and would seem to mediate different physiological responses to light [18]. Collectively these findings show that the pRGC subtypes are morphologically, anatomically and functionally distinct cell types, although their specific physiological functions remains to be fully determined. As the different retinal cell layers are not fully formed at birth and stratification of ganglion cell dendrites occurs postnatally [19], it is difficult to classify pRGCs that appear early in development as either M1 or M2 type pRGCs based upon morphology and localisation of dendrites alone. As such, little is known concerning the development of these functionally different cell types.

We have shown previously that two distinct isoforms of mouse melanopsin, Opn4L and Opn4S, are generated by alternative splicing of the murine *Opn4* gene [20]. These two isoforms of melanopsin differ only in their C-terminal regions and are differentially expressed in M1 and M2 type pRGCs in the adult mouse retina. M1 cells express both Opn4L and Opn4S whereas only Opn4L can be detected in M2 type cells. To date, the developmental expression of Opn4L and Opn4S has not been investigated, and as such it is not clear whether this differential pattern of expression is present in pRGCs from birth or occurs postnatally as specific pRGC subtypes develop. In this study we use qPCR and immunohistochemistry to investigate the expression of Opn4S and Opn4L isoforms during postnatal development of the mouse retina. Our results show a different profile of expression for Opn4L and Opn4S during development, and we propose that these differences are linked to the differential maturation of M1 and M2 subtypes of pRGCs.

## Methods

### Ethics Statement

All procedures were conducted in accordance with the Animals (Scientific Procedures) Act 1986 (PPL 70/6382 and 30/2812) and the University of Oxford Policy on the Use of Animals in Scientific Research and approved by the Home Office (UK) Animals Scientific Procedures Department (ASPD). All procedures were performed in a designated establishment. Animals were sacrificed by Schedule 1 methods. Retinal tissue from neonates (<P10) was collected following a non-schedule 1 method (decapitation), under authority of the relevant project licence.

### Animals

Wild type mice (C3H/He; not carrying *rd* mutation) [21] were housed under a 12∶12 LD cycle with food and water *ad libitum*. For analysis of developmental time points, animals (P0, P3, P5, P10, P14 and P30, n = 6 per time point) were sacrificed at ZT8 and eyes were removed and processed for immunohistochemistry or retina isolated and stored at −80°C for PCR analysis. For analysis of daily rhythms in melanopsin mRNA expression, C3H/He mice (P30–90) were sacrificed at ZT3, ZT8, ZT13, ZT18 and ZT23, n = 4 per time point. ZT0 = lights on, ZT12 = lights off.

### qPCR

Tissue was homogenised in TRIzol Reagent (Life Technologies) and total RNA isolated using RNeasy spin columns (Qiagen) and DNase treatment (Qiagen). RNA content was quantified using a nanandrop spectrophotometer (Thermo), and for each sample 0.5 µg of total RNA was reverse transcribed using SuperScript III with oligo(dT)_20_ primers (Life Technologies). Quantitative PCR was performed using Sybr Green I mastermix on a StepOne thermal cycler (Applied Biosystems) as described previously [20]. Relative quantification of transcript levels was performed with a comparative CT approach with levels of target gene expression normalised to the geometric mean expression of three house-keeping genes, *Gapdh*, *Psmb2* and *Arbp* [22]. Primer sequences are shown in Table 1.

**Table 1:** Primer Sequences.

Target	Forward Primer 5′-3′	Reverse Primer 3′-5′
*Opn4L*	GCTACCGCTCTACCCACC	CTACAGATGTCTGAGAGTCAC
*Opn4S*	GCTACCGCTCTACCCACC	CTACATCCCGAGATCCAGACT
*Rho*	TGTTCCTGCTCATCGTGCTGG	GGAAGTTGCTCATCGGCTTGC
*MWS*	ATGGTGGTGGTGATGGTCTTCG	TGTCTTGGAGGTGCTGGAAAGTTC
*TH*	TGCTGTTCTCAACCTGCTCTTCTCC	GGGTCTCTAAGTGGTGGATTTTGGC
*Per-2*	GGGGTGAGATTCGTCATTGAACTTG	AGGACATTGGCACACTGGAAAGAG
*c-fos*	ATCGGCAGAAGGGGAAAGTAG	GCAACGCAGACTTCTCATCTTCAAG
*GAPDH*	TGCACCACCAACTGCTTAG	GATGCAGGGATGATGTTC
*ARBP*	CGACCTGGAAGTCCAACTAC	ATCTGCTGCATCTGCTTG
*PSMB2*	AAATGCGGAATGGATATGAAT	GAAGACAGTCAGCCAGGTT

*Antibodies:* Polyclonal antibodies recognising the different C-terminal regions of murine Opn4L (raised in rabbit) and Opn4S (raised in goat) were raised against 15 amino acid synthetic peptides conjugated to KLH by Harlan UK, according to their standard procedures (Opn4S: SPQTKGHLPSLDLGM; Opn4L: PHPHTSQFPLAFLED) [20]. Both antibodies were affinity purified prior to use using standard techniques (Thiolink gel kit, Severn Biotech Ltd). A rabbit polyclonal antibody recognising the N-terminus of murine Opn4 common to both Opn4L and Opn4S was obtained commercially (UF006; Advanced Targeting Systems) [23].

### Validation of antibodies

All three melanopsin antibodies used in this study have been characterised previously, and label a subset of cells in the ganglion cell layer of the retina consistent with pRGCs in wildtype but not *Opn4^−/−^* mice [8], [10], [20], [23]. The Opn4L and Opn4S antibodies recognise only their respective isoforms [20], and the UF006 antibody recognises both Opn4L and Opn4S (Figure S1).

### Immunohistochemistry

Whole eyes (n = 4 animals for each time point) were removed, punctured with a fine gauge needle and placed in 4% paraformaldehyde (PFA) (Thermo Scientific) in PBS at 4°C for 16 h. Eyes were then cryoprotected in 30% sucrose in PBS at 4°C for 48 h, embedded in OCT medium (Sakura Finetek) and stored at −80°C. 18 µm tissue sections were cut at −23°C using a Leica CM1850 cryostat (Leica Microsystems) and collected on poly-L-lysine coated slides (Thermo Scientific). For immunohistochemistry, sections were permeabilised in PBS with 0.2% Triton-X for 20 mins at RT then blocked in PBS with 10% donkey serum for 1 h at RT. N-terminal UF006 (1∶2,500), Opn4S (1∶100) and Opn4L (1∶50) primary antibodies were incubated for 24–72 h at 4°C. Donkey anit-rabbit Alexa555 and donkey anti-goat Alexa488 secondary antibodies (Life Technologies) were incubated for 2 h at RT diluted 1∶200. All antibodies were diluted in PBS with 2.5% donkey serum and 0.2% Triton-X. All wash steps were performed using PBS 0.05% Tween-20. Sections were mounted with Prolong Gold anti-fade media containing DAPI (Life Technologies). Fluorescence images were acquired using an Olympus IX71 inverted microscope fitted with a high sensitivity CCD camera (Cascade 512B, Photmetrics) and Metamorph image acquisition software (Molecular Devices). Excitation filters for DAPI, green and red fluorescence were, 350 nm, 480 nm and 545 nm respectively. Emission filters were 460 nm, 525 nm and 600 nm.

### Statistical analysis

All data are shown ± standard error of mean and statistical analysis was performed using single factor ANOVA and two-tailed unpaired Student's t-Test.

## Results

### Expression of *Opn4L* and *Opn4S* mRNA during postnatal development

**Figure 1:**
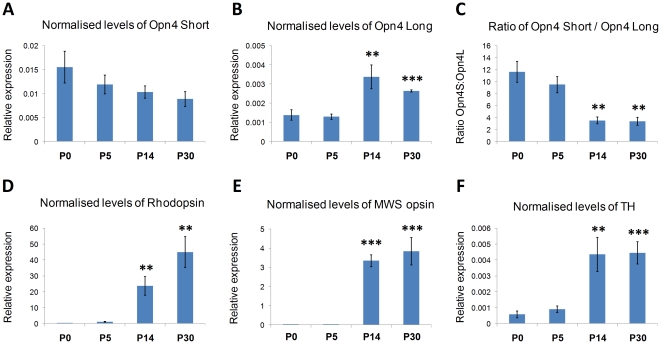
Expression of *Opn4L* and *Opn4S* mRNA during postnatal development. qPCR analysis showing the differential expression of Opn4S (A) and Opn4L (B) mRNA during postnatal development of the retina, highlighted by the changing ratio of Opn4L/Opn4S mRNA (C). Developmental profiles for rhodopsin (D), middle wave sensitive (MWS) cone opsin (E) and tyrosine hydroxylase (TH) are shown to indicate the timeline for the development of the classical visual system. All expression data is shown normalised to the expression of three housekeeping genes (Gapdh, Pmsb2, Arbp). Statistical results are shown for comparisons to data at P5. * = p<0.01, ** = p<0.001, *** = p<0.0001. For Opn4L, Opn4S and TH no significant differences were observed between P0 and P5, but significant increases were detected for rhodopsin (p = 0.001) and MWS (p = 0.02) when comparing these time points.

To investigate the developmental expression profiles of *Opn4L* and *Opn4S* we performed quantitative PCR using whole retina samples isolated at P0, P5, P14 and P30 (n = 6 per time point, all tissue collected at ZT8). PCR analysis showed a different expression profile for *Opn4L* and *Opn4S* transcripts during postnatal retinal development. Following normalisation to the expression of three housekeeping genes (*Gapdh*, *Arbp*, *Pmsb2*) levels of *Opn4S* remained relatively consistent across the developmental time points investigated, with a modest reduction in levels observed over time (ANOVA not significant, *F_(3,19)_* = 1.7, *P* = 0.20) (Figure 1A). In contrast normalised levels of *Opn4L* expression were low at P0 and P5 but showed a significant increase in expression (∼2.5 fold) at P14 and P30 (*F_(3, 19)_* = 10.0, *P* = 0.004, post hoc t-test, p = 0.006 and p = 4.1E-6 respectively) (Figure 1B). A direct comparison between *Opn4S* and *Opn4L* expression levels again showed a different profile of expression during postnatal development with a significant change in the ratio of *Opn4S* and *Opn4L* observed at P14 and P30 compared to earlier time points (Figure 1C) (*F_(3,20)_* = 14.1, *P* = 0.0001, p = 0.002 and p = 0.002). Consistent with the known timeline for the maturation of functional rod and cone photoreceptors [24], normalised levels of rhodopsin mRNA (F*_(3,19)_* = 14.4 , *P* = 0.0001, p = 0.002 and p = 0.001) and middle wave sensitive (MWS) cone opsin mRNA (*F_(3,19)_* = 33.5, *P* = 0.0001, p = 3.2E-7 and p = 0.0002) both showed a marked increase at P14 and P30 compared to P5 (Figure 1D and E). In addition levels of tyrosine hydroxylase mRNA (a marker of dopaminergic cells) also showed a significant increase at P14 and P30 time points (*F_(3,17)_* = 11.4, *P* = 0.0002, p = 0.007 and p = 0.0005) (Figure 1F). Neither Opn4L, Opn4S nor TH showed a significant increase between P0 and P5, but significant increases were detected for rhodopsin (p = 0.001) and MWS cone opsin (p = 0.02).

### Expression and localisation of Opn4L and Opn4S protein during postnatal development

**Figure 2:**
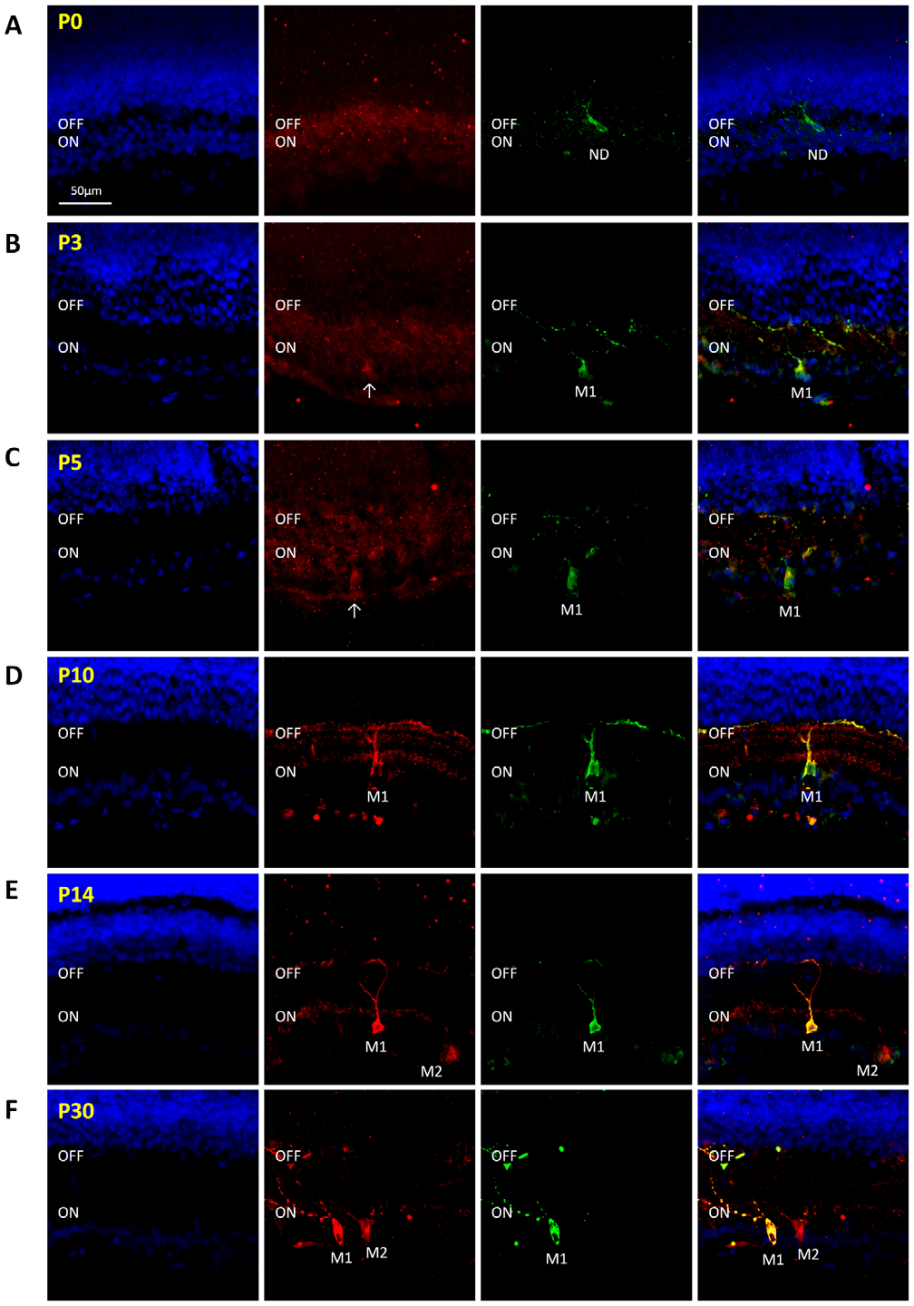
Expression of Opn4L and Opn4S protein during postnatal development. Immunohistochemistry images showing the expression of Opn4L (red) and Opn4S (green) throughout postnatal retinal development. Note the changing ratio of Opn4L and Opn4S staining from P0 to P30. Opn4S is detected at all time points, with levels of expression increasing from P0 to P3 but then rising only gradually until P10, whereby the majority of Opn4S positive cells clearly resemble M1 type pRGCs. Opn4L is not detected at P0, and only very weakly at P3 and P5 within cells that also express Opn4S (presumably M1 type pRGCs). Levels of Opn4L are slightly increased at P10 compared to P5, but are greatly increased at P14, with this rise coinciding with the widespread detection of cells resembling M2 type pRGCs that express only Opn4L. For all images DAPI nuclear counterstain is shown in blue. Merged images are show in right hand panels. ON and OFF correspond to the ON and OFF sublamina of the inner plexiform layer. M1 and M2 indicate cells classified as M1 and M2 type pRGCs. Arrows on images at P3 and P5 correspond to weakly Opn4L positive cells that can only be classified based on visualisation of Opn4S expression. Levels of brightness and contrast enhancement have been increased for P0–P5 Opn4L images to confirm the lack of detectable staining above background. Scale bar for all images = 50 µm.

**Figure 3:**
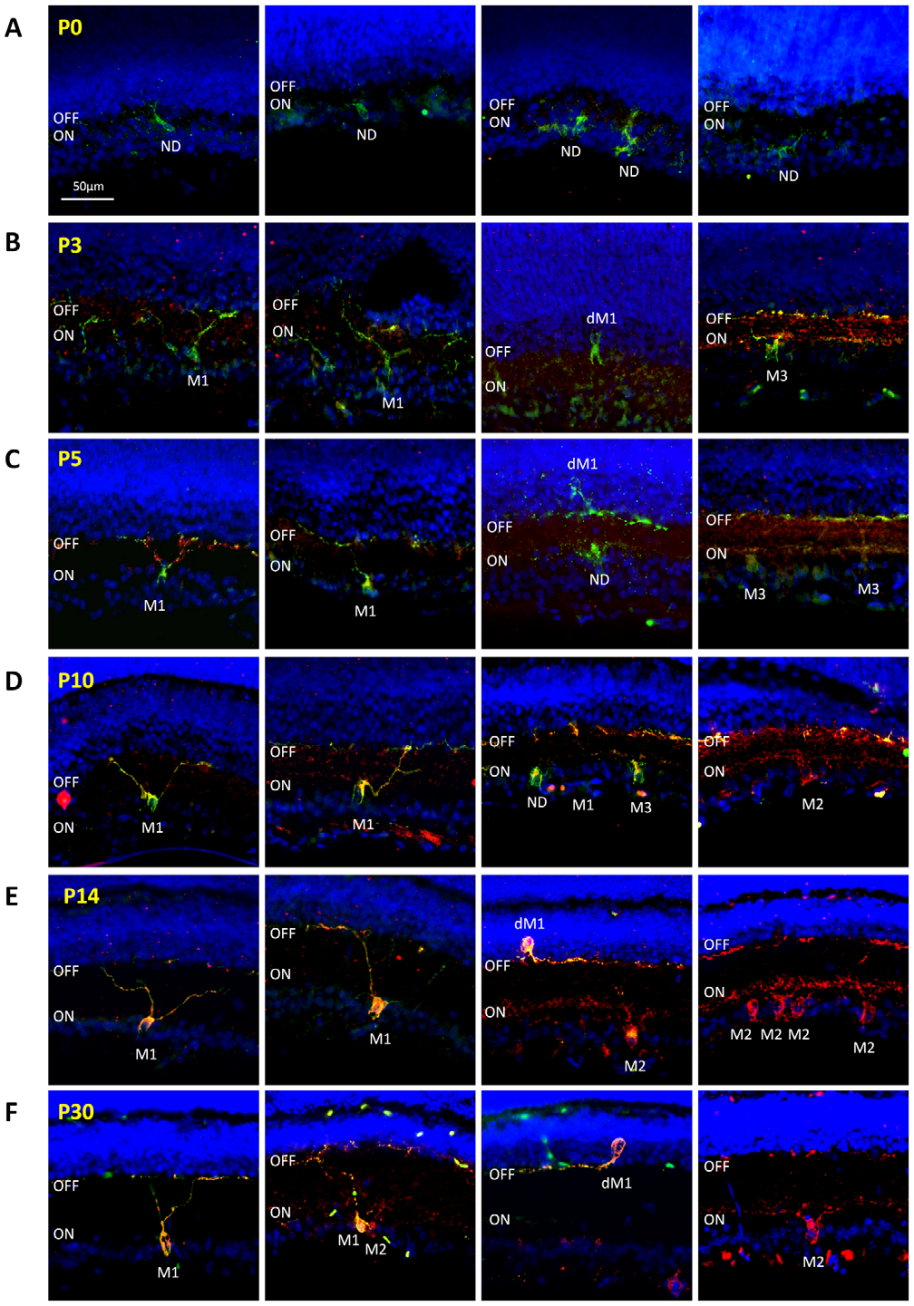
Differential expression and of Opn4L and Opn4S during development correlates with the maturation of M1 and M2 type pRGCs. Immunohistochemistry images showing the expression of Opn4L (red) and Opn4S (green) throughout postnatal retinal development, highlighting the variety of cell types detected at each time point. Note that Opn4L and Opn4S positive cells resembling M1 type pRGCs can be preliminarily identified as early as P3 and clearly be P5, whereas cells resembling M2 type pRGCs are detected only very weakly at P10 and not reliably detected until P14, following a large increase in levels of Opn4L staining. For all images DAPI nuclear counterstain is show in blue. ON and OFF correspond to the ON and OFF sublamina of the inner plexiform layer. M1 = M1 type pRGCs, M2 = M2 type pRGCs, dM1 = displaced M1 type pRGC, M3 = M3 type pRGC, but is also used to label cells that are multi stratified during early development, ND = not defined, for cells that could not be classified based on morphology alone. Scale bar for all images = 50 µm.

**Figure 4:**
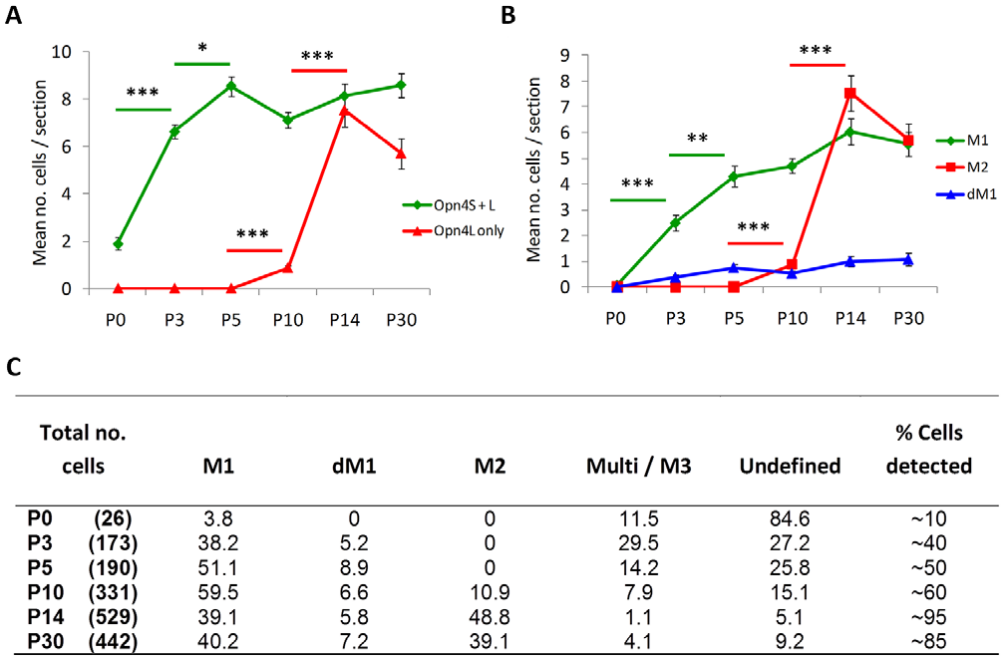
Summary of Opn4L and Opn4S expression profiles and timeline for maturation of M1 and M2 type pRGCs. A) Graph showing the mean number of cells in which Opn4S and Opn4L or only Opn4L were detected per retinal section at each development time point. Note the increasing numbers of Opn4S positive cells (also expressing Opn4L after P0) detected between P0 and P3 and also the increase in number of Opn4L only cells detected at P14 compared to earlier time points. B) Graph showing the mean number of cells from each retinal section that could be reliably classified as either M1, M2 or displaced M1 type pRGCs (dM1) based on morphology and stratification of dendrites. In all cases, cells classified as M1 and M2 type pRGCs were positive for Opn4S and Opn4L or Opn4L only respectively. Changes in immuno-positive cells were assessed against the previous time point in a stepwise manner. Significant differences are indicated, where the cell type is indicated by markers of the respective colours. This data demonstrates significant increases in Opn4S expression at P0–P5 and Opn4L at P5–14. Corresponding increases in the number of cells identified as M1 and M2 cells were observed at P0–P5, and P5–P14 respectively. * = p<0.05, ** = p<0.001, *** = p<1.0E-6. The overall correlation between the number of cells expressing Opn4S and Opn4L and those that could be classified as M1 type pRGCs based on morphology was 0.91. All cells expressing only Opn4L were classified as M2 type pRGCs. C) Summary of the classifications assigned to cells at each time point shown as a percentage of the total number of cells identified. As shown, cells resembling M1 type pRGCs were the dominant cell type identified in the early postnatal retina, with M2 type cells not detected reliably until P14. Number of sections examined was typically 25–30 per retina, from n = 3–4 retina per time point. Cells with dendrites located in multiple layers of the IPL or were clearly bistratified were classified as Multi / M3. Cells for which dendrites could not be clearly identified were classified as undefined. Prior to P10, all cells classified as undefined expressed both Opn4S and Opn4L (except at P0 where Opn4L could not be detected) and most likely represent early M1 type cells. In order to estimate the total number of cells that went undetected at each time point using the Opn4L and Opn4S antibodies (levels of under-reporting), the mean number of cells identified per retinal section using the isoform specific antibodies was compared to values observed from similar analysis of sections stained using the highly sensitive UF006 antibody that recognises both Opn4L and Opn4S.

Because of the significant changes in *Opn4L* mRNA observed between P5 and P14 additional time points were included for protein analysis. Immuno-staining of retinal sections from P0, P3, P5, P10, P14 and P30 retina (n = 3 retina per time point) with Opn4S and Opn4L isoform-specific antibodies again showed a differential pattern of expression for Opn4S and Opn4L during postnatal retinal development. Opn4S was detected at all time points investigated, with levels of Opn4S staining increasing steadily over time from P0–P10. By contrast Opn4L could not be detected at P0 and was detected only weakly in the early postnatal retina with a clear up-regulation observed between P10 and P14 (Figure 2 and Figure 3). Based on our observations this differing pattern of expression would seem to correlate with the maturation of M1 and M2 type pRGCs. A detailed description of our observations is given below and is summarised in Figure 4.

Opn4S expression was detected at P0, although levels of staining were low, with only a small number Opn4S expressing cells detected (Figure 4). The modest level of dendrite labelling and the lack of stratification observed at this time point prevent classification of these cells as either M1 or M2 type pRGCs. Despite repeated efforts, Opn4L could not be detected at P0 (Figure 2A and Figure 3A). By P3 the intensity of Opn4S labelling is increased in individual pRGCs and allows the detection of a significantly increased number of Opn4S positive cells (*F_(5, 151)_* = 10.6, *P*<0.0001, post hoc t-test, p = 1.2E-8). Extensive networks of dendritic processes are now visible, and are typically observed in all sublamina of the developing IPL (Figure 2B and Figure 3B). Low levels of Opn4L labelling were also observed at P3, although in all cases expression of Opn4L was detected only in cells in which Opn4S was also detected (Figure 2B and Figure 3B). Even at this early time point a number of cells (38.2%, 66 of 173 melanopsin positive cells) could be provisionally identified as early M1 type cells with major process terminating almost exclusively in the developing OFF layer of the IPL (Figure 2B and Figure 3B). By P5, levels of both Opn4S and Opn4L staining were moderately increased compared to P3, with a small further increase in the number of cells detected (p = 0.03). However again all melanopsin cells detected expressed both Opn4S and Opn4L with levels of Opn4S labelling remaining markedly higher than that of Opn4L. In general, the level of cellular process appear somewhat refined by P5 compared to P3, and although a notable proportion of pRGCs identified at P5 (Opn4S and Opn4L positive) remained multi-stratified (14.2%, 27 of 190 cells) or were otherwise unidentifiable as either M1 or M2 type cells, a large proportion of cells had morphologies clearly resembling early M1 type cells (51.1%, 97 of 190 cells) (Figure 2C and Figure 3C). Opn4S negative Opn4L positive cells, or cells otherwise resembling M2 type cells were not identified at P5 using these antibodies. By P10, levels of Opn4S and Opn4L labelling were again increased in individual cells. Cells with morphologies typical of mature M1 type cells were clearly identifiable. These cells represented the vast majority of cells detected at this time point (59.5%, 197 of 331 cells) and in all cases were positive for both Opn4S and Opn4L (Figure 2D and Figure 3D). A small percentage of multi-stratified or bistratified cells, expressing both Opn4S and Opn4L, were still detected at P10 although these were rarer than at P5 (7.9% and 14.2% respectively). In addition, at P10 cells expressing only Opn4L and resembling M2 type cells could also be identified. However, these cells were weakly labelled (barely detected above background) and rarely detected, representing 10.9% (36 of 331 cells) of all melanopsin positive cells identified at this time point (Figure 3D). At P14, cells with morphologies similar to both mature M1 and M2 type cells were clearly evident. Expression of both Opn4S and Opn4L was again detected in all cells resembling M1 type cells, with levels of melanopsin staining not noticeably increased above that seen at P10 (Figure 2E and Figure 3E). However, in contrast to P10, at P14 cells with morphologies resembling M2 type pRGCs were strongly labelled and easily identifiable (Figure 2E and Figure 3E). These cells express Opn4L alone, and represented 48.8% (258 of 529 cells) of all melanopsin positive cells detected, with the total number of Opn4L only cells detected now significantly increased compared to P10 (*F_(5, 151)_* = 59.2, *P*<0.0001, post hoc t-test, p = 1.2E-15). The dendritic processes of these Opn4L positive Opn4S negative M2 type cells were particularly pronounced at this time point, with a wide, densely packed band of processes evident in the ON layer of the IPL (Figure 2E and Figure 3E). This marked increase in Opn4L staining, and the increase in the number of detectable M2 cells coincides with the marked increase in expression of *Opn4L* mRNA that occurs between P5 and P14 (Figure 1B). At P30, cells with morphologies resembling mature M1 and M2 type cells were again detected, with M1 type cells (40.2%, 178 of 442 cells) expressing both Opn4S and Opn4L and M2 type cells (39.1%, 173 of 442 cells) expressing only Opn4L (Figure 2F and Figure 3F). For both M1 and M2 type cells, levels of melanopsin expression within individual pRGCs was broadly similar at P30 compared to that observed at P14. However, the density and branching complexity of dendrites observed for Opn4L expressing M2 cells appear markedly reduced at P30 compared to P14, with processes typically now stratifying in a narrow band of the ON layer of the IPL (Figure 2F and Figure 3F). The number of pRGCs that remained bistratified in P14 and P30 tissue was low (2–5% of all pRGCs). These bistratified cells, resembling M3 type pRGCs, were typically found to express both Opn4S and Opn4L. In addition to the classical M1 and M2 type cells we also observed a significant proportion of displaced melanopsin positive ganglion cells with their cell bodies located in the inner nuclear layer. The number of displaced pRGCs was relatively constant from P5 (8.9%) to P30 (7.5%) (Figure 4). Without exception all displaced pRGCs detected with these antibodies were positive for both Opn4S and Opn4L and had dendrites restricted to the OFF layer of the IPL, consistent with these cells being displaced M1 type pRGCs (Figure 3C, 3E and 3F).

Overall, the results of our ICC studies using the Opn4L and Opn4S specific antibodies show a different profile of Opn4L and Opn4S expression over time during development, and based on our observations this differing pattern of expression would seem to correlate with the maturation of M1 and M2 type pRGCs (where maturation is defined as cells reaching adult levels of melanopsin expression). Opn4S expression and cells resembling M1 type pRGCs are observed early in postnatal development, whereas in contrast the increase in Opn4L expression detected between P10 and P14 coincides with a marked increase in the detection of cells resembling M2 type pRGCs. Based on our observations, M2 cells cannot be detected until P10, and not reliably until P14 using the Opn4L antibody. One possible explanation is that M2 type cells are not present in the retina prior to this time and emerge later in development. Another, more likely explanation is that these cells are present prior to this time, but the lower levels of melanopsin expressed prevents detection by the Opn4L antibody. To address this suggestion, localisation of melanopsin protein in the developing retina was also performed using a highly sensitive melanopsin antibody raised against the N-terminal region of mouse melanopsin common to both Opn4L and Opn4S isoforms (UF006 antibody). Labelling with the UF006 antibody was notably more sensitive and intense compared to that observed for Opn4L and Opn4S antibodies and allowed for more accurate visualisation of cellular processes and improved detection of melanopsin positive cells. This was particularly evident in P0–P5 retina samples in which increased numbers of melanopsin expressing cells were detected compared to that seen with the isoform specific antibodies (Figure S2 and Figure S3). Estimates of the percentage of melanopsin expressing cells detected using the isoform specific antibodies at each time point is shown in Figure 4C. In agreement with the results obtained with the Opn4S and Opn4L antibodies, staining of retina sections and whole retina flatmounts with the UF006 antibody again suggests a different timeline for the maturation of M1 and M2 type pRGCs (Figure S2 and Figure S3).

**Figure 5:**
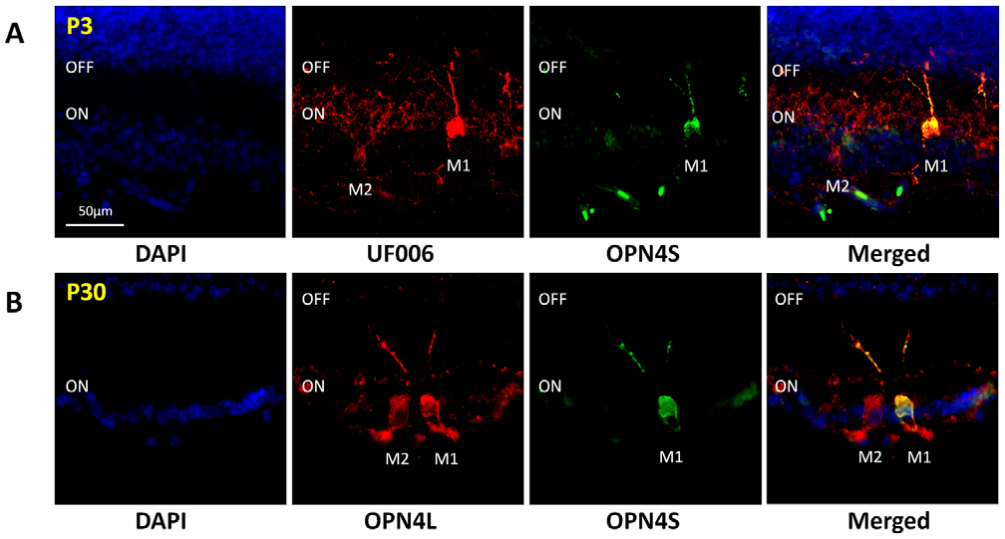
Differential expression of Opn4S is observed early in postnatal development in cells resembling M1 and M2 type pRGCs. A) Double labelling with UF006 (red) and Opn4S (green) antibodies showing the differential expression of Opn4S in M1 and M2 type pRGC subtypes as early as P3. B) Double labelling with Opn4L (red) and Opn4S (green) antibodies at P30 showing the morphology and pattern of melanopsin expression in M1 and M2 type pRGCs of the adult retina. Note the similarities in morphology and levels of staining for M1 type pRGCs observed at P3 and P30, compared to the changes in morphology and staining that are observed for M2 type cells between these time points. For all images DAPI nuclear counterstain is show in blue. Merged images are show in right hand panels. Scale bar for all images = 50 µm.

Due to the increased sensitivity of the UF006 antibody, Opn4S negative cells resembling early M2 type cells could be identified as early as P3, although levels of staining were typically low (Figure 5A). At this time point M2 cells have dense dendritic fields that occupy a much broader area of the ON layer of the IPL than that observed at P30 (Figure 5B). In comparison, the morphology ad stratification of M1 cells at P3 are much more similar to that observed in adult tissue (Fig. 5A and 5B). Due to the weaker Opn4S (and Opn4L) antibody staining, and the low number of Opn4S positive cells that can be detected in the P0 retina, the vast majority of cells detected at P0 were positive only for UF006 (and not Opn4S or Opn4L). It was therefore not possible to accurately determine the isoforms of melanopsin expressed in the majority of pRGCs at P0.

### Daily rhythm in *Opn4L* and *Opn4S* mRNA expression

**Figure 6:**
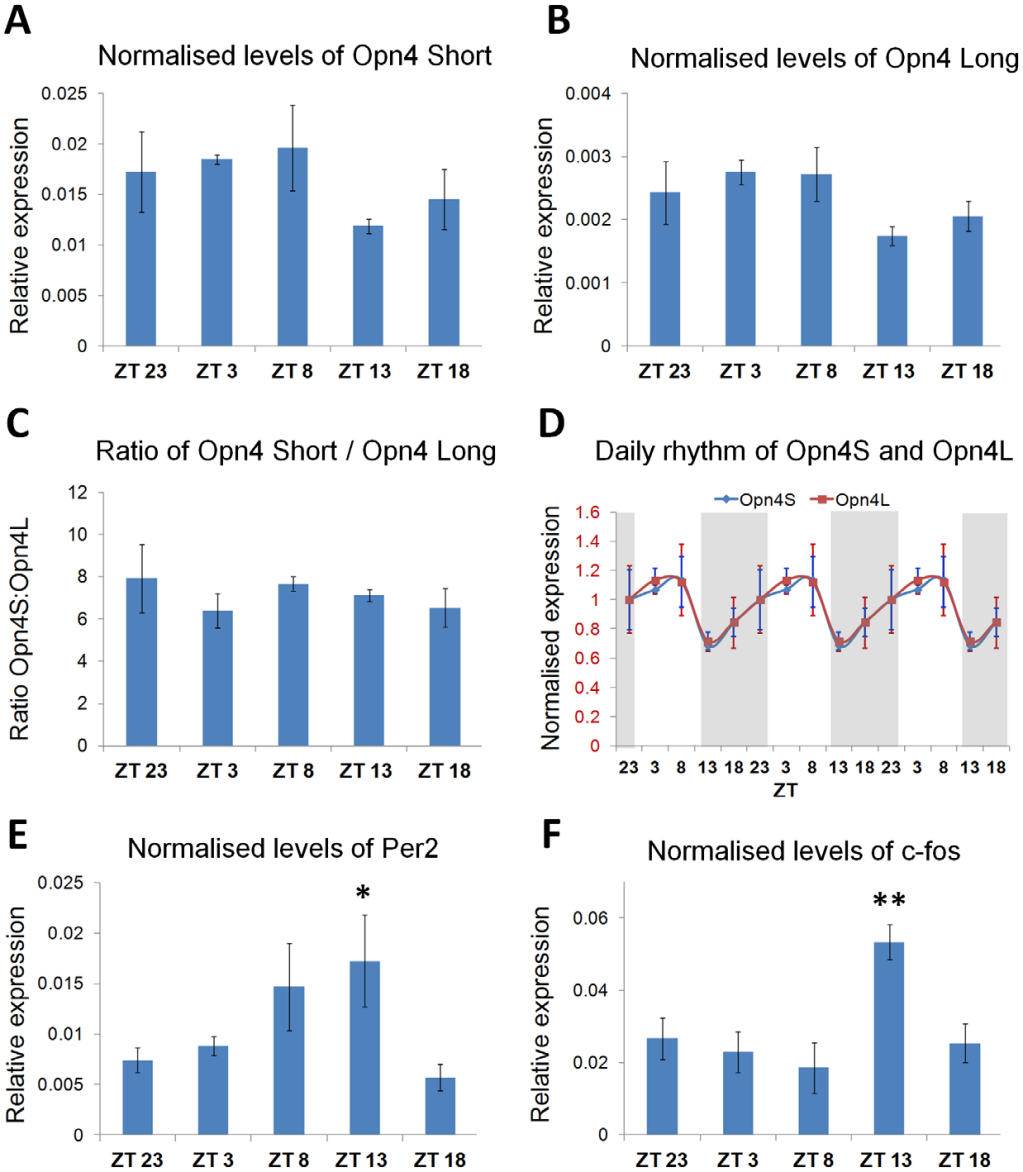
Daily rhythm of *Opn4L* and *Opn4S* mRNA expression in the adult retina. qPCR analysis showing the levels of *Opn4S* (A) and *Opn4L* (B) mRNA expression detected at different ZT time points from adult mice (P30–60) housed under 12∶12 light dark cycles. ZT0 = light on, ZT12 = lights off. C) Graph showing the ratio of *Opn4S*∶*Opn4L* mRNA detected at different ZT time points. Note that data is triple plotted to highlight the rhythmic trend. Periods of light and dark are indicated by shaded regions. D) Comparison of normalised daily rhythms for *Opn4L* and *Opn4S*. E) Levels of *Per2* and F) Levels of *c-fos* mRNA expression detected at different ZT time points. All data is shown normalised to the expression of three housekeeping genes (*Gapdh*, *Pmsb2*, *Arbp*). Statistical significance is shown for post-hoc t-tests for comparisons to ZT23. * = p = 0.05, ** = p<0.01.

To investigate the potential effect of daily rhythms in melanopsin expression we examined the profile of both *Opn4L* and *Opn4S* mRNA expression at different times throughout the day in adult mice (>P30) housed under 12∶12 light dark cycles. Results of qPCR analysis revealed no significant difference in levels of either *Opn4L* mRNA (*F_(4,20)_* = 2.13, *P* = 0.13) or *Opn4S* mRNA (*F_(4,20)_* = 1.31, *P* = 0.31) at any time point investigated (Figure 6). Although not significant, the results of our qPCR analysis do suggest a subtle trend for both *Opn4L* and *Opn4S*. The rhythms of expression of both *Opn4S* and *Opn4L*, if present, would appear to be synchronised, with no significant change in the ratio of *Opn4S*∶*Opn4L* detected at any time point investigated (*F_(4,20)_* = 0.13, *P* = 0.97). Rhythmic patterns of expression were detected for both *Per2* mRNA (*F_(4,20)_* = 2.60, *P* = 0.082, p = 0.0506) and *c-fos* mRNA (*F_(4,20)_* = 5.86, *P* = 0.006, p = 0.007) in these samples, consistent with previous studies [25], [26]. However despite the obvious trend for *Per2* mRNA expression we failed to observe any significant differences for this data based on ANOVA analysis, although post-hoc t-Test analysis does suggest a significant difference between ZT13 and ZT18 (p = 0.0506).

## Discussion

The broad aims of this study were to determine the expression of the recently identified Opn4 isoforms, *Opn4L* and *Opn4S*, during postnatal retinal development. The results described here indicate that Opn4L and Opn4S are differentially expressed during the development of the mouse retina, and that the differential expression of these melanopsin isoforms may be linked to the functional maturation of M1 and M2 type pRGCs.

### Opn4L and Opn4S are differentially expressed throughout development

Levels of *Opn4S* mRNA expression were relatively constant throughout development. By contrast levels of *Opn4L* mRNA were low at birth and showed a significant (∼2.5 fold) up-regulation at P14 and P30 compared to earlier time points. In all likelihood the absolute levels of both *Opn4S* and *Opn4L* transcripts (i.e. total number of copies) are increased over time, yet this increase is masked by the process of normalising target gene expression to the levels of housekeeping gene expression, where the increasing number of cells in the early postnatal retina [27] will result in a dilution of melanopsin mRNA as a percentage of the total mRNA pool. However, it is clear that the changes in expression of *Opn4L* are more marked than those of *Opn4S*, an observation confirmed by the changing ratio of *Opn4S*/*Opn4L* detected at P14 and P30 compared to earlier time points. Given the similarities in the amplification efficiencies for the *Opn4L* and *Opn4S* primers used in this study [20] it would seem that despite the increase in *Opn4L* expression at P14, *Opn4S* remains the dominant transcript expressed in the retina throughout the stages of postnatal development. Analysis of Opn4S and Opn4L protein expression confirmed the results of our PCR studies, and again demonstrated a differential expression of these isoforms during postnatal development. Opn4S protein was detected at all time points with a sharp rise detected between P0 and P3, followed by a more gradual increase over time until P10 where adult levels were observed. By contrast, levels of Opn4L were low in the early retina and showed a large up-regulation between P10 and P14.

### Differential expression of Opn4L and Opn4S in development correlates with the developmental maturation of M1 and M2 type pRGCs

Consistent with our previous findings [20], we report a differential pattern of expression for Opn4L and Opn4S within subtypes of pRGCs in the adult retina. At P14 and P30, where pRGC subtypes can be easily classified based on stratification of their dendrites in the ON and OFF layers of the IPL, cells identified as M1 type pRGCs were found to express both Opn4S and Opn4L whereas only Opn4L could be detected in M2 type pRGCs. Our current results now demonstrate a differential pattern of expression for both Opn4S and Opn4L during postnatal retinal development and suggest that this difference in profile of expression may be linked to the differential maturation of M1 and M2 type pRGCs. Based on our observations, M1 cells would seem to mature first, with Opn4S detected at P0, and Opn4S and Opn4L expressing cells with morphologies resembling M1 cells provisionally identified as early as P3, and more clearly by P5, with mature M1 type morphology clearly evident by P10. By contrast, Opn4L positive M2 type cells with processes confined to the ON layer of the IPL could not be detected at P0, P3 or P5 using the Opn4L antibody, and were only rarely detected at P10. However, by P14 Opn4L positive M2 cells are strongly labelled and represent around 50% of all melanopsin positive cells identified. Overall, the increase in *Opn4L* mRNA expression observed at P14 coincides with a large increase in the overall levels of Opn4L protein expression and an increase in the number of M2 type pRGCs that can be detected. We suggest that the increase in photopigment expression that occurs in M2 type pRGCs may correlate with a functional maturation of these cells, most likely resulting in an increase in endogenous photosensitivity and function. This is in contrast to M1 type cells that have detectable levels of melanopsin expression from birth, show a rapid increase in levels of expression between P0 and P3, and reach adult levels of melanopsin expression earlier, between P5 and P10. Based on these data, Opn4S is the dominant photopigment present at birth and M1 type pRGCs would appear to be the dominant cell type during early postnatal development, and are likely to be responsible for the earliest light detection events that occur in the mammalian retina.

It is possible that some of the cells classified as M2 type pRGCs in this study may correspond to the recently identified M4 and M5 type pRGCs that also have processes located in the ON layer of the IPL [8]. We think this unlikely, however, as levels of melanopsin expression are reportedly very low in these cells and they are not easily identified with the UF006 antibody but instead are visualised only via detection of a fluorescent reporter in a transgenic mouse line [8], [10]. It is therefore unlikely that we would detect these cells using the less sensitive Opn4L or Opn4S antibodies.

Limitations in the sensitivity of immunohistochemistry prevent us from determining whether the differential expression of Opn4S and Opn4L occurs in cells of the P0 retina, and as such we are unable to determine whether or not early pRGCs are predisposed to forming M1 and M2 type cells prior to birth or whether these cells differentiate postnatally from a common ‘melanopsin pre-cursor’ cell. However, our results from double labelling with UF006 and Opn4S antibodies suggests that differential expression of Opn4S is present in pRGCs as early as P3, and as such it would seem that cellular specialisation of pRGCs occurs early, and before the process of stratification is completed.

In general the findings of our expression studies are consistent with previous reports. Levels of melanopsin are known to be relatively low at birth, and show a marked increase during the first few days of postnatal development [4], [17], [28], [29], resulting in a significant increase in the photosensitivity of individual pRGCs [4], [12], [28]. We would suggest that these early changes in melanopsin expression are consistent with the increased levels of Opn4S expression observed between P0 and P3 in our study and most likely represents the functional maturation of M1 type pRGCs. A further increase in sensitivity of pRGC responses is observed around P11, driven largely by the emergence of functional rod and cone photoreceptors [12]. It is currently unclear whether or not there is also an increase in the levels of intrinsic photosensitivity in M2 type pRGCs around this time point, as would be expected from our data.

### Why do M1 and M2 type pRGCs mature at different times during development – a functional link between M2 type cells and the classical visual system?

The reason why M1 and M2 pRGC subtypes mature at different times during development is not clear. It is interesting to note that the time frame for the up-regulation of Opn4L mRNA and protein, and the apparent maturation of M2 type pRGCs occurs at a similar time to the up-regulation of rhodopsin, MWS cone opsin and tyrosine hydroxylase mRNA and coincides with the maturation of the classical visual system. In the adult retina pRGCs are known to receive irradiance information from rod and cone photoreceptors via synapses with bipolar and amacrine cells and act as the principle conduit for passing rod and cone driven information to brain areas involved in non-image forming responses to light [30], [31], [32], [33]. However, rod and cone photoreceptors are not functional at birth but develop postnatally, and do not become fully functional until P10–12 [34], [35] with eye opening typically observed around P13–14. Consistent with this timeline, pRGCs begin to receive rod cone driven light signals at around P11, resulting in significant changes in the cellular responses of individual pRGCs [12]. From this it is clear that the melanopsin system, although active from birth cannot become fully mature until the classical rod and cone photoreceptors have emerged and functional connections are made between melanopsin cells and the outer retina. There is now emerging evidence that the function of M2 type cells may be more dependent on rod and cone input than are M1 type cells. M2 type cells express much lower levels of melanopsin protein and are less endogenously photosensitive than M1 type cells, eliciting only small photocurrents in response to light stimulation in the absences of rod cone driven input [8], [14], [36]. In addition, recent studies have also shown that inputs from cone ON bipolar cells generate large depolarising currents in M2 type cells, but only small responses in M1 type cells [15]. Collectively these data suggest that the rod and cone pathway exerts more influence on the functioning of M2 cells than M1 cells. The photoresponses of M2 type cells are driven primarily by excitatory inputs from the ON pathway with relatively little contribution from melanopsin phototransduction, whereas melanopsin function is almost entirely responsible for the generation of photoresponses in M1 type cells [15]. Based on these observations, it seems possible that the development or maturation of M2 type pRGCs may be timed to more closely coincide with the maturation of the classical visual system.

### Daily rhythms in melanopsin expression

A number of studies have indicated that light exposure, or exposure to light dark cycles, can influence levels of melanopsin expression, potentially via the involvement of dopaminergic amacrine cells [37], [38], [39], [40], [41]. It is therefore possible that daily rhythms in Opn4L and Opn4S expression may contribute to the different profiles of expression that we observe throughout development. For example, Gonzalez-Menendez et al. [39] have reported a daily rhythm in the number of melanopsin expressing cells that can be detected via immunostaining in mice housed under 12∶12 light dark cycles. These results suggest a significant decrease in the number of M1 cells detected between ZT23 and ZT3 at the transition from dark to light, and a more subtle increase in the number of M2 cells detected from ZT18 to ZT23. This rhythm of melanopsin protein expression is absent under conditions of constant darkness, suggesting a light driven effect rather than an intrinsic circadian rhythm of melanopsin expression in the adult retina [39]. Despite these previous reports, we could not detect a significant daily rhythm in expression of either Opn4L or Opn4S mRNA in adult mice housed under identical conditions to those reported previously by Gonzalez-Menendez et al. [39]. Our data did however suggest a trend, and it is possible that a higher number of replicate samples may have allowed detection of a subtle rhythm. However, based on our results, should daily rhythms in expression of Opn4L and Opn4S exist in the adult retina it would seem that they are synchronised, with no evidence of a daily variation in the ratio of *Opn4S* and *Opn4L* mRNA detected. It is however possible that the process of analysing levels of *Opn4L* and *Opn4S* mRNA from the whole retina has masked subtle rhythms that may exist within the specific subsets of pRGCs.

From our data, it is clear that the magnitude of daily changes detected for *Opn4L* and *Opn4S* mRNA in the adult retina are relatively small. Should daily rhythms of similar magnitude exist during development, it is unlikely that changes in the phase of these rhythms could explain the marked changes in Opn4L expression, and detection of M2 cells that we observe between P10 and P14. Dopamine is known to be light regulated in the retina [42], [43], and dopaminergic amacrine cells (DACs) have been implicated in the regulation of melanopsin expression [40], [41]. Given the close interactions of DACs and M1 type pRGCs [32] it would seem that dopamine exerts influence primarily on M1 type cells, a conclusion supported by recent findings [41]. However, given the lack of functional rods, cones and dopamine amacrine cells, we could realistically expect light dark cycles to exert less influence on melanopsin expression in the early postnatal retina. In support of this conclusion, it has been shown that light exposure exerts no effect on the increased levels of melanopsin expression that occur in the first 24 hours after birth [29]. Daily variations in melanopsin expression have been reported for M1 cells as early as P5, although rhythms of expression are seemingly absent from M2 cells at this time point [39]. Based on these reports, if daily rhythms of expression are influencing the results of this study we could reasonably expect larger variations in the expression of Opn4S compared to Opn4L. However, in comparison to the significant changes we detect for Opn4L, levels of Opn4S show no such significant changes in expression between any developmental time points investigated. Overall, it would seem there is little compelling evidence for a robust daily rhythm in melanopsin expression within M2 cells in either the adult or postnatal retina [39]. Given these observations, it would seem unlikely that daily variations in melanopsin expression can explain the different profiles of Opn4L and Opn4S expression that we detect during development, or explain the dramatic increase in detection of Opn4L and M2 type pRGCs between P10 and P14. Furthermore, daily variations in melanopsin expression would not explain the clear difference in timeline for the morphological maturation of the pRGC subtypes that we observe. As such, we believe that a differential maturation of M1 and M2 type pRGCs best explains the differential profile of Opn4L and Opn4S expression detected in this study.

In support of our general conclusions regarding the differential maturation of M1 and M2 type pRGCs, McNeill et al. [17] have recently reported that different populations of pRGCs are born at different times during prenatal development, and also that different pRGC subtypes innervate their specific brain targets at different time points during postnatal development [17]. These data again suggest that the neural circuitry associated with the different melanopsin systems do indeed mature at different rates during postnatal development. It is possible that the up regulation of melanopsin expression within specific subsets of pRGCs may be a direct result of these cells establishing functional connections with their respective recipient brain targets, or the onset of functional activity in these target structures. Future studies will be required to investigate these possibilities and determine that factors that regulate expression of the different melanopsin isoforms and the postnatal development of specific pRGC subtypes.

### Summary

In summary, the results of the present study demonstrate that Opn4L and Opn4S are differentially expressed during postnatal development, at both mRNA and protein levels. Our data suggest a model whereby there are two distinct phases of increased melanopsin expression during postnatal development, an initial increase between P0 and P3 driven primarily by an increase in Opn4S expression, and a second increase that occurs between P10 and P14 driven by changes in Opn4L expression. Based on our observations these two phases of increased melanopsin expression would seem to correlate with the functional maturation of M1 and M2 type pRGCs. We suggest that the specific functions associated with M1 and M2 type pRGCs will mature at different times during postnatal development.

## Supporting Information

Figure S1 **N-terminal melanopsin antibody UF006 recognises both Opn4L and Opn4S.** Representative images showing positive staining of both Opn4L and Opn4S transfected Neuro-2A cells following incubation with the UF006 N-terminal melanopsin antibody. Transient transfection and antibody staining of Neuro-2A cell cultures was performed as described previously [20].
(TIF)

Figure S2 **UF006 flatmount staining throughout postnatal development.** Representative images collected from whole mount retina stained with the N-terminal melanopsin antibody (UF006). Note the initial increase in melanopsin staining observed between P0 and P3 and also the increase in weakly stained M2 type pRGCs observed at P14 compared to earlier time points. Based on cell counts from whole flat mounted retina, we detected 1600–1800 pRGCs per adult retina (P30), comprising approximately 45% M1 type cells and 55% M2 type pRGCs, values that are consistent with previous estimates of total numbers of melanopsin cells in the adult mouse retina [10]. M1 = M1 type pRGCs, M2 = M2 type pRGCs, dM1 = displaced M1 type pRGC, M3 = M3 type pRGC, but is also used to label cells that are multi stratified during early development, ND = not defined, for cells that could not be classified based on morphology alone. For staining of whole retina flatmounts, whole eyes were collected and fixed as described for retinal sections. Retinae were then dissected and cryoprotected in 30% sucrose prior to freeze thaw cycles (×2) with liquid N_2_. Retina were then washed in PBS with 1% Triton-X ×3 for 10 mins, blocked in 10% donkey serum with 1% Triton-X for 1 h at RT, and incubated with UF006 (1∶2500) for 72 h at 4°C, and Alexa 555 secondary antibody (1∶200) for 24 h at 4°C, both diluted in PBS with 2% donkey serum and 1% Triton-X.
(TIF)

Figure S3 **Melanopsin expression during postnatal development.** Representative images showing the pattern of melanopsin expression throughout postnatal retinal development as detected using the UF006 melanopsin antibody (recognises both Opn4L and Opn4S). At P0 levels of melanopsin expression are low, with the majority of pRGCs only weakly labelled and not typically identifiable as either M1 or M2 type pRGCs based on morphology alone. Levels of melanopsin expression are markedly increased by P3, and cells with morphologies resembling M1 type pRGCs are tentatively identified at this time point, and more definitively by P5 at which point the intensity of staining was similar to that seen in adult tissue. Cells with morphologies resembling M2 type pRGCs were tentatively identified at P3 and more easily by P5 but levels of staining was low (presumably too low for detection with the Opn4L antibody). A marked increase in the intensity of staining was observed for M2 type pRGCs by P14. At P14 M2 type cells are strongly labelled and typically have extensive dense networks of processes spanning a relatively broad region of the ON sublamina of the IPL. By P30 the processes of M2 type cells appear less pronounced and typically stratify in a narrow band of the ON layer of the IPL, consistent with the results of the isoform specific antibodies. DAPI nuclear counterstain is show in blue. ON and OFF correspond to the ON and OFF sublamina of the inner plexiform layer. M1 = M1 type pRGCs, M2 = M2 type pRGCs, dM1 = displaced M1 type pRGC, M3 = M3 type pRGC, but is also used to label cells that are multi stratified during early development, ND = not defined, for cells that could not be classified based on morphology alone. Scale bar for all images = 50 µm.
(TIF)
