# Supplementary figures and images for: Differential Expression of Melanopsin Isoforms Opn4L and Opn4S during Postnatal Development of the Mouse Retina
Source: PLoS One. 2012 Apr 5;7(4):e34531. doi: 10.1371/journal.pone.0034531 (PMC3320640; doi:10.1371/journal.pone.0034531)

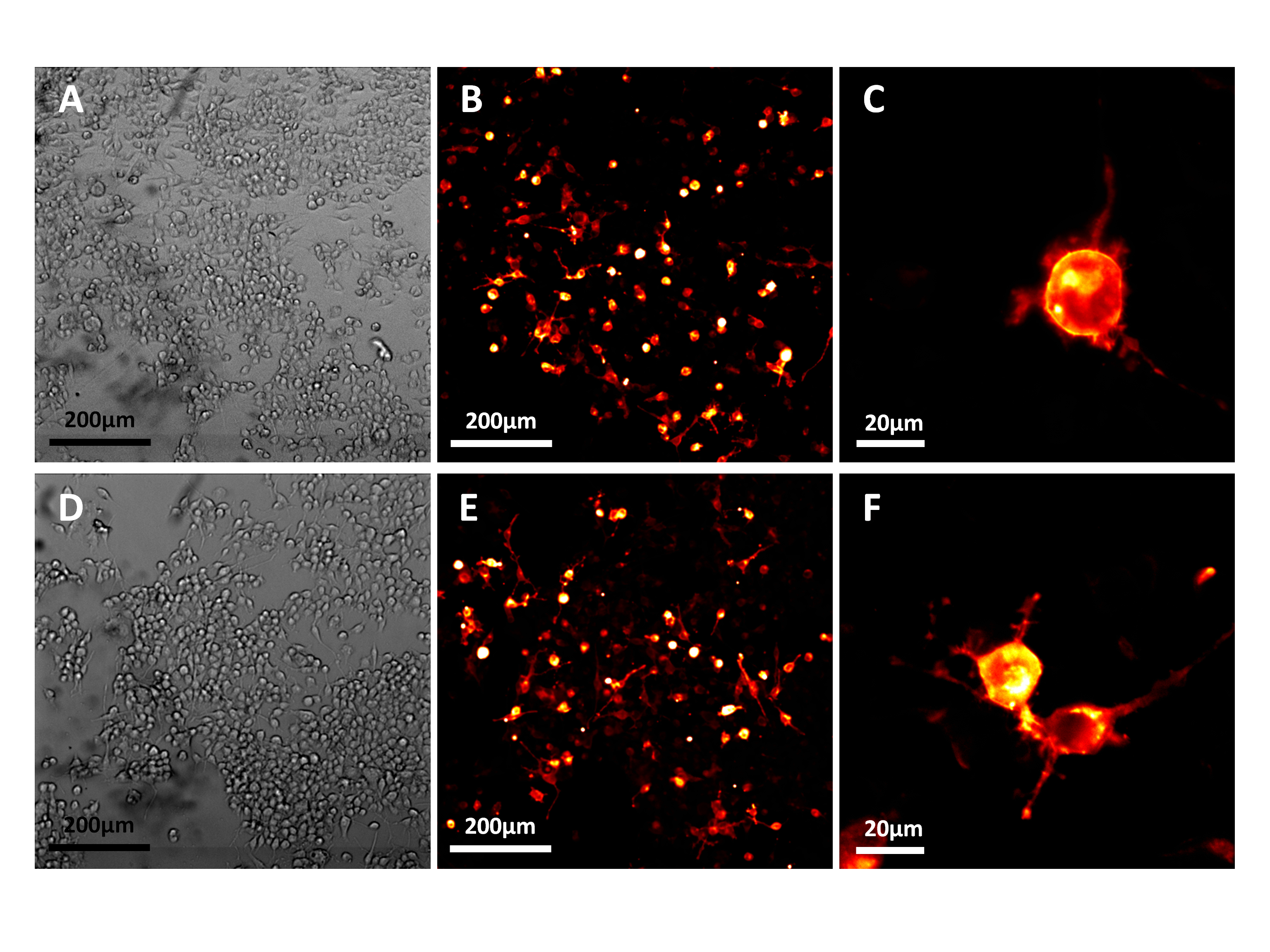

Supplement: Figure S1 — N-terminal melanopsin antibody UF006 recognises both Opn4L and Opn4S. Representative images showing positive staining of both Opn4L and Opn4S transfected Neuro-2A cells following incubation with the UF006 N-terminal melanopsin antibody. Transient transfection and antibody staining of Neuro-2A cell cultures was performed as described previously [20]. (TIF) [file pone.0034531.s001.tif]

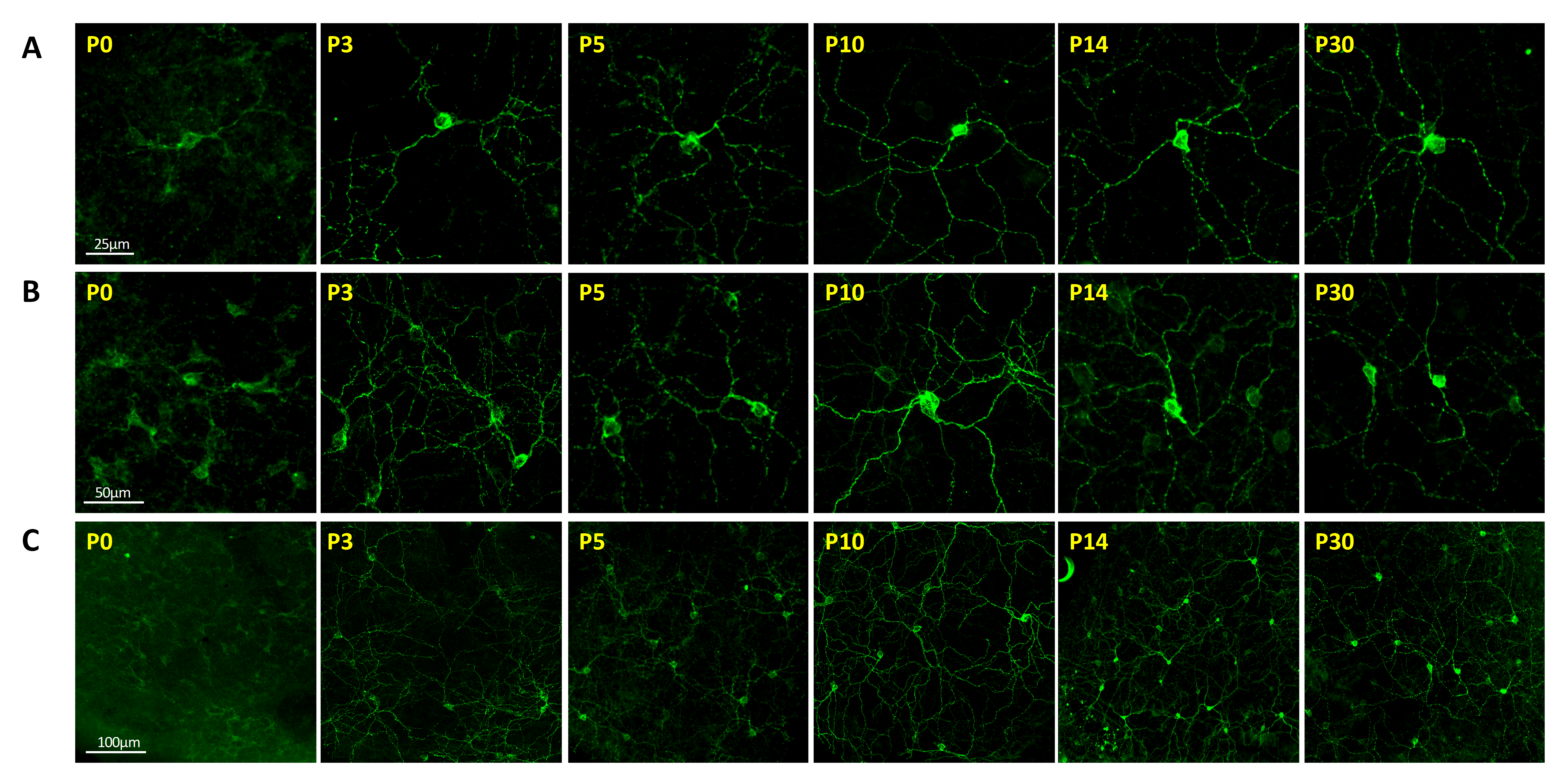

Supplement: Figure S2 — UF006 flatmount staining throughout postnatal development. Representative images collected from whole mount retina stained with the N-terminal melanopsin antibody (UF006). Note the initial increase in melanopsin staining observed between P0 and P3 and also the increase in weakly stained M2 type pRGCs observed at P14 compared to earlier time points. Based on cell counts from whole flat mounted retina, we detected 1600–1800 pRGCs per adult retina (P30), comprising approximately 45% M1 type cells and 55% M2 type pRGCs, values that are consistent with previous estimates of total numbers of melanopsin cells in the adult mouse retina [10]. M1 = M1 type pRGCs, M2 = M2 type pRGCs, dM1 = displaced M1 type pRGC, M3 = M3 type pRGC, but is also used to label cells that are multi stratified during early development, ND = not defined, for cells that could not be classified based on morphology alone. For staining of whole retina flatmounts, whole eyes were collected and fixed as described for retinal sections. Retinae were then dissected and cryoprotected in 30% sucrose prior to freeze thaw cycles (×2) with liquid N2. Retina were then washed in PBS with 1% Triton-X ×3 for 10 mins, blocked in 10% donkey serum with 1% Triton-X for 1 h at RT, and incubated with UF006 (1∶2500) for 72 h at 4°C, and Alexa 555 secondary antibody (1∶200) for 24 h at 4°C, both diluted in PBS with 2% donkey serum and 1% Triton-X. (TIF) [file pone.0034531.s002.tif]

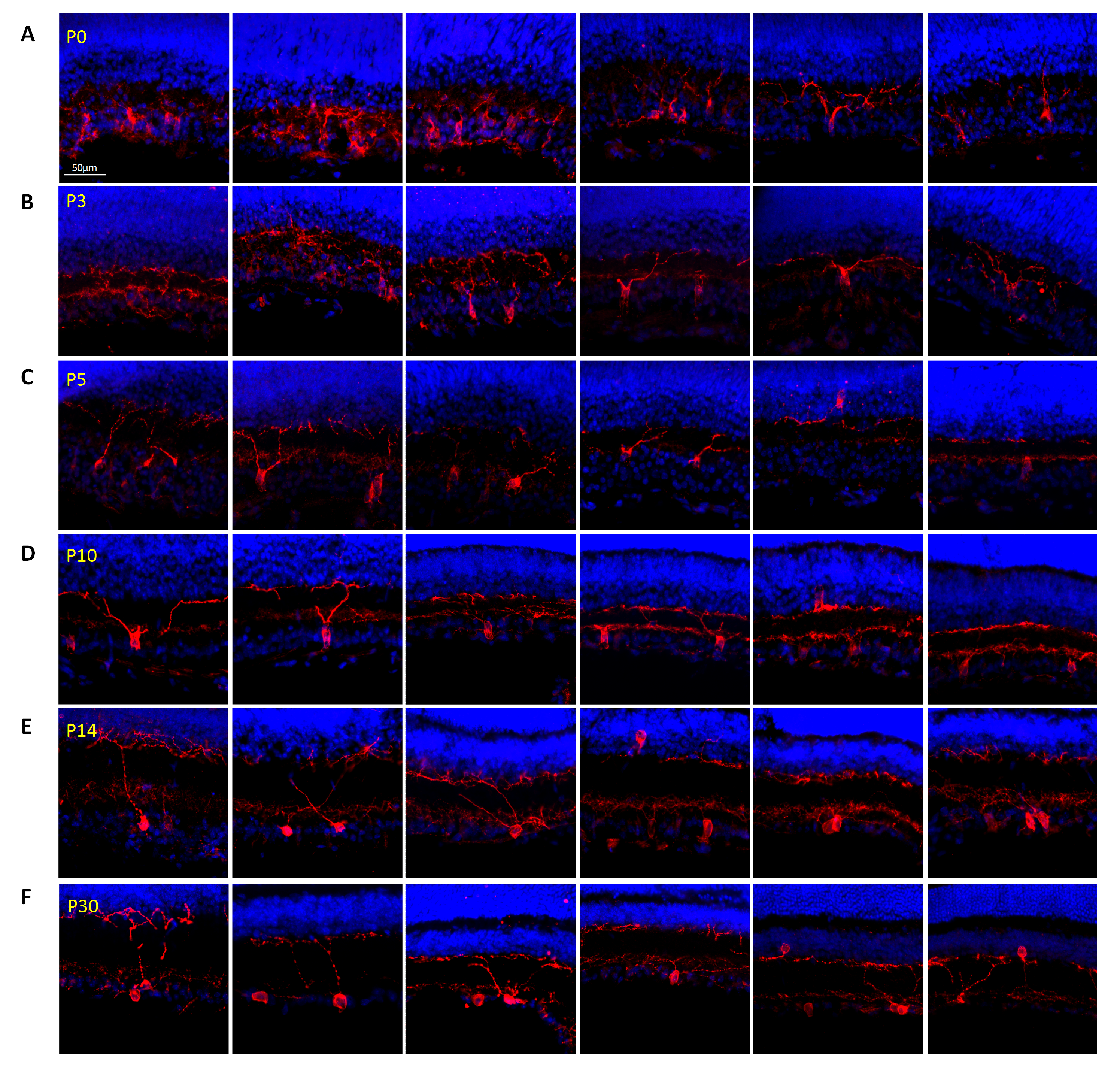

Supplement: Figure S3 — Melanopsin expression during postnatal development. Representative images showing the pattern of melanopsin expression throughout postnatal retinal development as detected using the UF006 melanopsin antibody (recognises both Opn4L and Opn4S). At P0 levels of melanopsin expression are low, with the majority of pRGCs only weakly labelled and not typically identifiable as either M1 or M2 type pRGCs based on morphology alone. Levels of melanopsin expression are markedly increased by P3, and cells with morphologies resembling M1 type pRGCs are tentatively identified at this time point, and more definitively by P5 at which point the intensity of staining was similar to that seen in adult tissue. Cells with morphologies resembling M2 type pRGCs were tentatively identified at P3 and more easily by P5 but levels of staining was low (presumably too low for detection with the Opn4L antibody). A marked increase in the intensity of staining was observed for M2 type pRGCs by P14. At P14 M2 type cells are strongly labelled and typically have extensive dense networks of processes spanning a relatively broad region of the ON sublamina of the IPL. By P30 the processes of M2 type cells appear less pronounced and typically stratify in a narrow band of the ON layer of the IPL, consistent with the results of the isoform specific antibodies. DAPI nuclear counterstain is show in blue. ON and OFF correspond to the ON and OFF sublamina of the inner plexiform layer. M1 = M1 type pRGCs, M2 = M2 type pRGCs, dM1 = displaced M1 type pRGC, M3 = M3 type pRGC, but is also used to label cells that are multi stratified during early development, ND = not defined, for cells that could not be classified based on morphology alone. Scale bar for all images = 50 µm. (TIF) [file pone.0034531.s003.tif]
